# Detection and Characterization of Bat Sarbecovirus Phylogenetically Related to SARS-CoV-2, Japan

**DOI:** 10.3201/eid2612.203386

**Published:** 2020-12

**Authors:** Shin Murakami, Tomoya Kitamura, Jin Suzuki, Ryouta Sato, Toshiki Aoi, Marina Fujii, Hiromichi Matsugo, Haruhiko Kamiki, Hiroho Ishida, Akiko Takenaka-Uema, Masayuki Shimojima, Taisuke Horimoto

**Affiliations:** The University of Tokyo, Tokyo, Japan (S. Murakami, T. Kitamura, M. Fujii, H. Matsugo, H. Kamiki, H. Ishida, A. Takenaka-Uema, T. Horimoto);; Yamaguchi University, Yamaguchi, Japan (J. Suzuki);; Iwate University, Iwate, Japan (R. Sato, T. Aoi);; National Institute of Infectious Diseases, Tokyo, Japan (M. Shimojima)

**Keywords:** betacoronavirus, bat sarbecovirus, Rhinolophus cornutus, respiratory infections, severe acute respiratory syndrome coronavirus 2, SARS-CoV-2, SARS, COVID-19, coronavirus disease, zoonoses, viruses, coronavirus, Japan

## Abstract

Epidemiology of bat *Betacoronavirus,* subgenus *Sarbecovirus* is largely unknown, especially outside China. We detected a sarbecovirus phylogenetically related to severe acute respiratory syndrome coronavirus 2 from *Rhinolophus cornutus* bats in Japan. The sarbecovirus’ spike protein specifically recognizes angiotensin-converting enzyme 2 of *R. cornutus*, but not humans, as an entry receptor.

During the past 20 years, coronaviruses belonging to the genus *Betacoronavirus* have caused multiple human epidemic or pandemic diseases, including severe acute respiratory syndrome (SARS), Middle East respiratory syndrome (MERS), and coronavirus disease (COVID-19). Two viruses of the subgenus *Sarbecovirus* are severe acute respiratory syndrome coronavirus (SARS-CoV), which causes SARS, and SARS-CoV-2, which causes COVID-19. Although *Rhinolophus* spp. bats in Asia, Europe, and Africa are considered natural reservoirs of sarbecoviruses (*1*–*3*), the epidemiology and distribution of these viruses remain largely unknown, especially outside China. Previously, partial RNA-dependent RNA polymerase (RdRp) genes of betacoronaviruses were detected in little Japanese horseshoe bats (*Rhinolophus cornutus*) (*4*). However, limited sequence information left the genetic and virological properties unclear. We detected and determined the entire genome sequence of a bat sarbecovirus belonging to a phylogenetic clade that includes SARS-CoV-2 from *R. cornutus* bats in Japan. Further, we used a pseudotyped virus system to characterize an entry step of this virus into cells.

## The Study

*R. cornutus* is an endemic bat species in Japan and is found nationwide. These bats primarily inhabit caves and abandoned tunnels in the countryside during daytime and capture insects at night outside their roosts. *R. cornutus* bats often cohabit with other insectivorous bats, such as *R. ferrumequinum* or *Myotis macrodactylus*, and occasionally with wild animals, such as the masked palm civet (*Paguma larvata*), in their daytime roosts. 

In 2013, we captured 4 *R. cornutus* bats in a cave in Iwate prefecture, Japan, and extracted RNA from fresh feces. Then, we used real-time reverse transcription PCR (rRT-PCR) to detect the partial RdRp gene of sarbecovirus from 2 samples by using a pair of primers designed to detect betacoronavirus. In 2020, we performed RNA sequencing and determined the full genome sequence of 1 sample, Rc-o319, which exhibited lower cycle threshold value by rRT-PCR.

**Figure 1:**
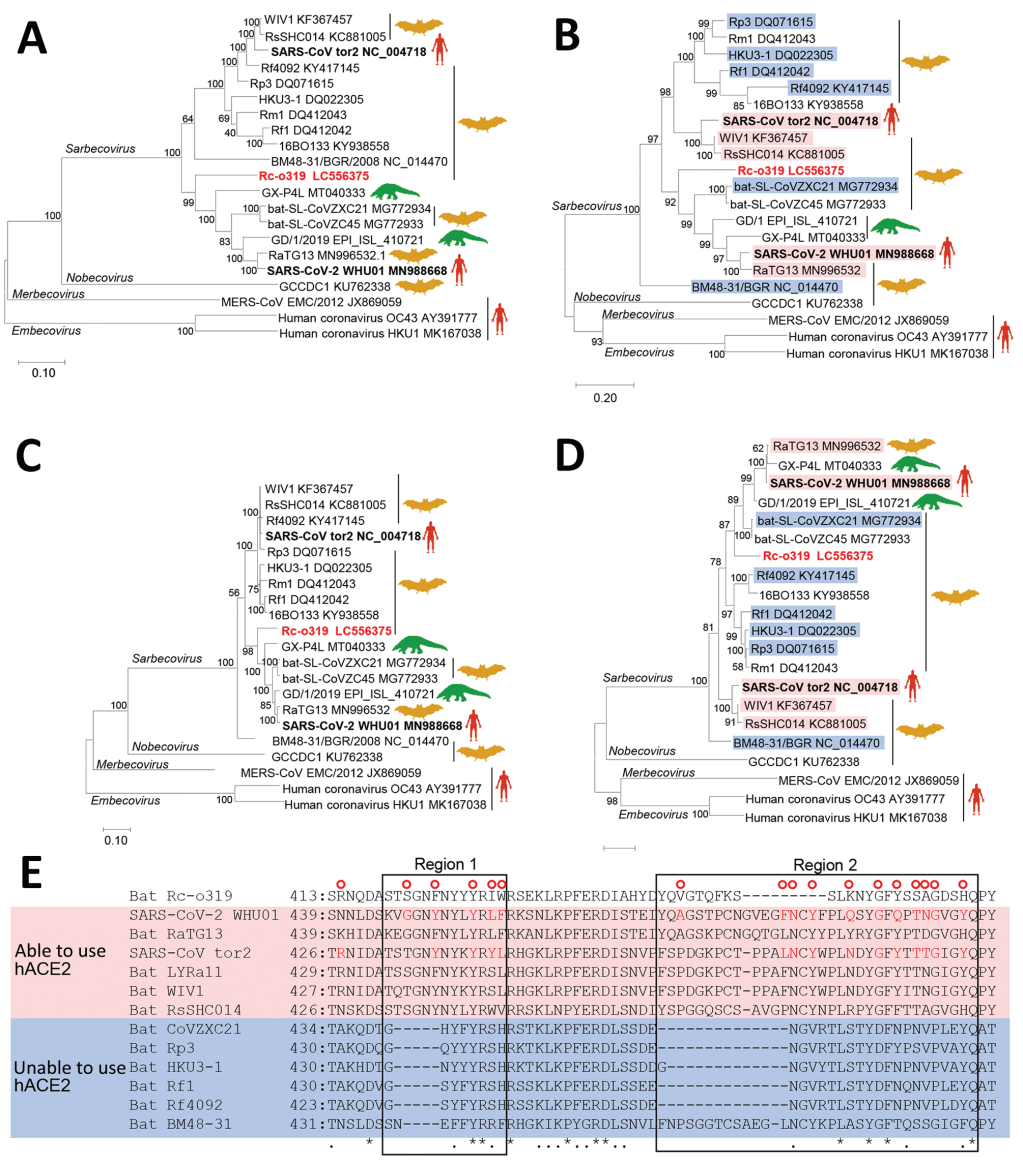
Phylogenetic analysis of sarbecovirus sequenced from little Japanese horseshoe bats (*Rhinolophus cornutus*) and genetically related to human SARS-CoV-2, Japan. A–D) Phylogenetic trees were generated by using maximum-likelihood analysis combined with 500 bootstrap replicates and show relationships between bat-, human-, and pangolin-derived sarbecoviruses. Phylogenetic trees are shown for nucleotide sequences of the full genome (A), the S protein gene and amino acid sequences (B), the ORF1ab (C), and the S protein (D). Red text indicates positions of Rc-o319, the sarbecovirus sequenced in this study. For panels B and D, magenta bands indicate viruses with S proteins that bind to human ACE2; blue bands indicate viruses with S proteins that do not bind to human ACE2. Bootstrap values are shown above and to the left of the major nodes. Scale bars indicate nucleotide or amino acid substitutions per site. E) Amino acid sequence alignment of the RBM of S proteins that are able or unable to bind to human ACE2. Amino acid residues of the RBM that contact human ACE2 of SARS-CoV-2 and SARS-CoV are indicated in the upper side by red circles. The 2 regions of S protein RBM known to interact with human ACE2 are indicated by boxes labeled region 1 and region 2. ACE2, angiotensin-converting enzyme 2; hACE2, human angiotensin-converting enzyme 2; ORF1ab, open reading frame 1ab; RBM, receptor-binding motif; S, spike protein; SARS-CoV, severe acute respiratory syndrome coronavirus; SARS-CoV-2, severe acute respiratory syndrome coronavirus 2.

We performed a BLAST (https://blast.ncbi.nlm.nih.gov/Blast.cgi) analysis of the full genome of Rc-o319, which showed Rc-o319 had the highest nucleotide homology to SARS-CoV-2 HKG/HKU-904a/2020 strain (GenBank accession no. MT365032) with a query cover of 96% and sequence identity of 81.47%. The maximum-likelihood analysis with sarbecoviruses demonstrated that the full genome and spike protein (S) gene of Rc-o319 were positioned within a specific clade that included SARS-CoV-2 (Figure 1, panels A, B). Amino acid sequences of open reading frame 1ab (ORF1ab) and S of Rc-o319 also were positioned within the SARS-CoV-2 clade (Figure 1 panels C and D). The phylogenetic trees maintained the same topology between ORF1ab and S, indicating that no recombination event occurred in Rc-0319, which was supported by similarity plot analysis (Appendix Figure 1). The nucleotide and amino acid sequences of Rc-o319 were more homologous to those of viruses belonging to SARS-CoV-2 clade than the SARS-CoV clade (Table). These data suggest that Rc-o319 genetically is related to SARS-CoV-2.

**Table:** Identities of nucleotide and amino acid sequences of genome, genes, and proteins to representative sarbecoviruses used to investigate sarbecovirus Rc-o319 detected in bats, Japan

Virus	Entire genome	ORF1ab	S	ORF3a	E	M	ORF6	ORF7a	ORF7b	ORF8	N	ORF10
	Nucleotide %											
SARS-CoV-2	81.5	80.0	73.0	83.2	97.4	86.6	86.6	78.4	77.3	53.3	88.3	94.9
RaTG13	81.2	79.8	73.3	83.9	96.9	85.4	87.1	77.4	78.0	53.0	87.8	94.0
pangolin/P4L	80.4	79.8	72.5	83.5	97.8	85.5	85.5	74.5	–	53.2	86.8	91.5
CoVZXC21	80.4	80.2	72.5	79.9	96.1	86.0	83.3	75.1	–	50.8	85.9	53.9
SARS-CoV-1	81.0	78.8	73.6	76.6	93.1	87.0	77.1	73.4	74.8	–	86.1	63.3
Rf1	80.6	78.7	70.9	75.1	92.6	85.8	78.8	72.2	75.6	68.6	84.9	63.3
BM48–31	79.6	77.4	69.3	70.4	90.5	80.6	61.3	65.1	57.9	–	77.0	64.1
	Amino acid, %											
SARS-CoV-2	NA	88.2	76.7	87.0	98.7	91.0	83.6	73.8	69.8	27.5	89.5	87.2
RaTG13	NA	88.1	77.6	86.6	98.7	91.0	83.6	73.0	72.1	28.2	89.7	84.6
pangolin/P4L	NA	88.2	76.9	86.2	98.7	91.0	80.3	68.9	–	28.8	89.2	79.5
CoVZXC21	NA	87.9	75.5	83.3	98.7	91.4	77.1	68.9	–	20.5	88.1	28.2
SARS-CoV-1	NA	85.9	75.2	72.5	93.4	97.7	66.7	69.7	68.2	–	88.9	59.0
Rf1	NA	85.5	73.6	69.6	92.1	94.1	66.7	68.0	72.7	66.7	87.4	59.0
BM48–31	NA	83.7	71.9	64.5	93.4	88.1	48.5	52.5	58.1	–	85.9	61.5
*NA, not available; ORF, open reading frame; –, no ORFs found.												

To gain insight into the zoonotic potential of Rc-o319, we focused on angiotensin-converting enzyme 2 (ACE2) receptor binding motif (RBM) of the S protein (Figure 1, panel E). RBM has 2 regions (1 and 2); both are essential to human ACE2 (hACE2) recognition (*5*,*6*). Several S proteins of bat-origin sarbecoviruses that lack the ability to bind the hACE2 contain amino acid deletions in both regions (Figure 1, panel E) (*5*). However, the RBM of Rc-o319 S is unique because it has 9 aa deletions in region 2 only, which was not observed in other bat sarbecoviruses. Of note, most residues that contact hACE2, which were detected in the S protein of SARS-CoV-2 and SARS-CoV, were different or missing in the S protein of Rc-o319. Thus, current data do not enable inference for whether the Rc-o319 can use hACE2 as a cell entry receptor.

**Figure 2:**
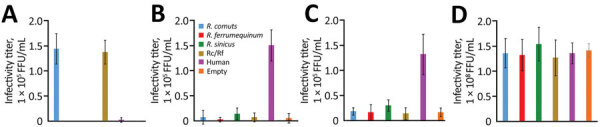
Infectivity titers of sarbecoviruses from bats and humans used to investigate bat sarbecovirus Rc-o319, which is genetically related to human SARS-CoV-2, Japan. Cells expressing each host-origin angiotensin-converting enzyme 2 were inoculated with VSV pseudotyped with spike proteins of Rc-o319 (A), SARS-CoV (B), SARS-CoV-2 (C), or glycoprotein of VSV (D). At 20 hour postinfection, GFP-positive cells were counted and the infectivity titers were calculated. Error bars indicate SDs from 3 independent experiments. CoV, coronavirus; GFP, green fluorescent protein; Rc/Rf, chimera of *Rhinolophus cornutus* and *R. ferrumequinum*; SARS, severe acute respiratory syndrome; VSV, vesicular stomatitis virus.

To evaluate the potential of ACE2 as a receptor for Rc-o319, we adopted a pseudotyped vesicular stomatitis virus (VSV) system, in which VSV glycoprotein envelope (G) gene is replaced by green fluorescent protein (GFP) gene. We generated VSV pseudotyped with S proteins of Rc-o319 (VSV-Rc-o319), SARS-CoV (VSV-SARS), SARS-CoV-2 (VSV-SARS-2), or VSV-G (VSV-VSV-G) in human embryonic kidney 293T (HEK293T) cells. We also constructed ACE2 expression plasmids from hACE2, *R. ferrumequinum* bats (Rf-ACE2), and *R. sinicus* bats (Rs-ACE2). *R. ferrumequinum* is another bat species inhabiting in Japan, and *R. sinicus* bats are a major host reservoir of bat sarbecoviruses. We also prepared a chimeric bat ACE2 from *R. cornutus* and *R. ferrumequinum* bats, Rc/Rf chimera, which has the N terminus of S protein interaction domain of Rc-ACE2 and the remaining region from Rf-ACE2 (Appendix Figure 2). The HEK293T cells expressing Rc-ACE2, Rf-ACE2, Rs-ACE2, Rc/Rf-ACE2, or hACE2 were produced by transfecting each ACE2-expression plasmid (Appendix Figure 3) and inoculating them with pseudotyped VSVs; their infectivity was titrated by counting GFP-positive cells at 20 hours postinfection (Figure 2). Our results showed that VSV-Rc-o319 infected Rc-ACE2– and Rc/Rf-ACE2–expressing cells, but not Rf-ACE2–, Rs-ACE2–, or hACE2–expressing cells or control cells transfected with empty vector plasmid. In contrast, VSV-SARS and VSV-SARS-2 more effectively infected hACE2-expressing cells than bat ACE2–expressing cells and control cells. VSV-VSV-G infected all tested cells to comparable levels, confirming ACE2-independent infectivity of VSV. These results suggest high specificity of ACE2 receptor between sarbecovirus and host cells and possibly a limited zoonotic potential of Rc-o319 in terms of cell receptor usage without adaptation to humans.

We next analyzed the membrane fusion step of Rc-o319 S. A previous study showed that human sarbecovirus S protein was proteolytically activated by cellular transmembrane serine protease 2 (TMPRSS2), in vitro and in vivo, inducing efficient virus-cell membrane fusion at the cell surface (*7*). We prepared a fusion assay, in which HEK293T cells were cotransfected with S-expression plasmid of Rc-o319, SARS-CoV, or SARS-CoV-2 and Rc-ACE2–, Rf-ACE2–, Rs-ACE2–, or hACE2-expression plasmid, together with fluorescent reporter Venus-expression plasmid with and without TMPRSS2-expression plasmid, and incubated for 24 h to assess syncytium formation. We observed that the S protein of SARS-CoV and SARS-CoV-2 required both hACE2 and TMPRSS2 for fusion activity (Appendix Figure 4), confirming the previous findings (*7*,*8*). In contrast, the S protein of Rc-o319 induced cell fusion only in Rc-ACE2–expressing cells, both in the presence and absence of TMPRSS2. These results suggest that unlike human sarbecoviruses, Rc-o319 uses Rc-ACE2 as a functional receptor, leading to membrane fusion independent of S-cleavage by TMPRSS2.

## Conclusions

Among *R. cornutus* bats in Japan, we detected sarbecovirus Rc-o319, which is phylogenetically positioned in the same clade as SARS-CoV-2. Sarbecoviruses belonging to this clade previously were detected from other *Rhinolophus* spp. bats and pangolins (family Manidae) in China and could have played a role in the emergence of SARS-CoV-2 (*9*–*11*). We provide a hypothesis that a bat sarbecovirus with zoonotic potential might exist even outside China, because *Rhinolophus* spp. bats inhabit Asia, Europe, and Africa (*12*).

Receptor usage is one factor for cross-species transmission of viruses. Unlike a previous report that showed that some bat SARS-CoV–related viruses could use human and civet ACE2 and *R. sinicus* ACE2 as entry receptors (*5*,*13*,*14*), VSV-Rc-o319 was found to use only homologous Rc-ACE2, which arguably suggests that Rc-o319 and its related viruses might not jump the species barrier easily and cause infection. However, Rc-o319 and its related viruses could be transmitted accidentally from *R. cornutus* bats to cohabitant animals, such as civets, which are potential intermediate hosts for human infection (*15*). Therefore, further epidemiologic surveillance of bat betacoronaviruses, including evaluation of their zoonotic potential, is essential because betacoronaviruses that caused SARS, MERS, and COVID-19 outbreaks in humans during the past 20 years likely originated from bat betacoronaviruses.

Appendix Additional methods used for detection and characterization of bat sarbecovirus phylogenetically related to severe acute respiratory syndrome coronavirus 2, Japan.

## References

[1] Drexler JF, Gloza-Rausch F, Glende J, Corman VM, Muth D, Goettsche M, et al. Genomic characterization of severe acute respiratory syndrome-related coronavirus in European bats and classification of coronaviruses based on partial RNA-dependent RNA polymerase gene sequences. J Virol. 2010;84:11336–49. 10.1128/JVI.00650-10 20686038 PMC2953168

[2] Hu B, Zeng LP, Yang XL, Ge XY, Zhang W, Li B, et al. Discovery of a rich gene pool of bat SARS-related coronaviruses provides new insights into the origin of SARS coronavirus. PLoS Pathog. 2017;13:e1006698. 10.1371/journal.ppat.1006698 29190287 PMC5708621

[3] Tao Y, Tong S. Complete genome sequence of a severe acute respiratory syndrome-related coronavirus from Kenyan bats. Microbiol Resour Announc. 2019 8(28):e00548–19. 10.1128/MRA.00548-19 PMC6624766 31296683

[4] Suzuki J, Sato R, Kobayashi T, Aoi T, Harasawa R. Group B betacoronavirus in rhinolophid bats, Japan. J Vet Med Sci. 2014;76:1267–9. 10.1292/jvms.14-0012 24871548 PMC4197156

[5] Letko M, Marzi A, Munster V. Functional assessment of cell entry and receptor usage for SARS-CoV-2 and other lineage B betacoronaviruses. Nat Microbiol. 2020;5:562–9. 10.1038/s41564-020-0688-y 32094589 PMC7095430

[6] Lan J, Ge J, Yu J, Shan S, Zhou H, Fan S, et al. Structure of the SARS-CoV-2 spike receptor-binding domain bound to the ACE2 receptor. Nature. 2020;581:215–20. 10.1038/s41586-020-2180-5 32225176

[7] Glowacka I, Bertram S, Müller MA, Allen P, Soilleux E, Pfefferle S, et al. Evidence that TMPRSS2 activates the severe acute respiratory syndrome coronavirus spike protein for membrane fusion and reduces viral control by the humoral immune response. J Virol. 2011;85:4122–34. 10.1128/JVI.02232-10 21325420 PMC3126222

[8] Yamamoto M, Kiso M, Sakai-Tagawa Y, Iwatsuki-Horimoto K, Imai M, Takeda M, et al. The anticoagulant nafamostat potently inhibits SARS-CoV-2 S protein-mediated fusion in a cell fusion assay system and viral infection in vitro in a cell-type-dependent manner. Viruses. 2020;12:E629. 10.3390/v12060629 32532094 PMC7354595

[9] Zhou P, Yang XL, Wang XG, Hu B, Zhang L, Zhang W, et al. A pneumonia outbreak associated with a new coronavirus of probable bat origin. Nature. 2020;579:270–3. 10.1038/s41586-020-2012-7 32015507 PMC7095418

[10] Lau SKP, Luk HKH, Wong ACP, Li KSM, Zhu L, He Z, et al. Possible bat origin of severe acute respiratory syndrome coronavirus 2. Emerg Infect Dis. 2020;26:1542–7. 10.3201/eid2607.200092 32315281 PMC7323513

[11] Zhang T, Wu Q, Zhang Z. Probable pangolin origin of SARS-CoV-2 associated with the COVID-19 outbreak. Curr Biol. 2020;30:1346–1351.e2. 10.1016/j.cub.2020.03.022 32197085 PMC7156161

[12] Stoffberg S, Jacobs DS, Mackie IJ, Matthee CA. Molecular phylogenetics and historical biogeography of Rhinolophus bats. Mol Phylogenet Evol. 2010;54:1–9. 10.1016/j.ympev.2009.09.021 19766726

[13] Ge XY, Li JL, Yang XL, Chmura AA, Zhu G, Epstein JH, et al. Isolation and characterization of a bat SARS-like coronavirus that uses the ACE2 receptor. Nature. 2013;503:535–8. 10.1038/nature12711 24172901 PMC5389864

[14] Yang XL, Hu B, Wang B, Wang MN, Zhang Q, Zhang W, et al. Isolation and characterization of a novel bat coronavirus closely related to the direct progenitor of severe acute respiratory syndrome coronavirus. J Virol. 2015;90:3253–6. 10.1128/JVI.02582-15 26719272 PMC4810638

[15] Guan Y, Zheng BJ, He YQ, Liu XL, Zhuang ZX, Cheung CL, et al. Isolation and characterization of viruses related to the SARS coronavirus from animals in southern China. Science. 2003;302:276–8. 10.1126/science.1087139 12958366

