# Supplementary material for: Detection and Characterization of Bat Sarbecovirus Phylogenetically Related to SARS-CoV-2, Japan
Source: Emerg Infect Dis. 2020 Dec;26(12):3025–9. doi: 10.3201/eid2612.203386 (PMC7706965; doi:10.3201/eid2612.203386)
Supplement: Appendix — Additional methods used for detection and characterization of bat sarbecovirus phylogenetically related to severe acute respiratory syndrome coronavirus 2, Japan. [file 20-3386-Techapp-s1.pdf]

Article DOI: https://doi.org/eid2612/203386

Detection and Characterization of Bat Sarbecovirus Phylogenetically Related to SARS-CoV-2, Japan

Appendix

Additional Methods

Sample Collection

We captured 4 *Rhinolophus cornutus* bats in a cave in Iwate prefecture, Japan, in 2013, with permission from the prefectural local government. Each bat was kept in a separate plastic bag. Fresh feces samples were collected, transferred into tubes containing sterilized saline, and frozen in dry ice. We released the bats after feces collection.

Reverse Transcription-PCR

RNA was extracted from the feces samples using an RNeasy PowerMicrobiome Kit (QIAGEN, https://www.qiagen.com). Next, we detected the partial RNA-dependent RNA polymerase (RdRp) gene of sarbecovirus in 2 samples by real-time reverse transcription-PCR by using RNA-direct SYBR Green Realtime PCR Master Mix (TOYOBO, https://www.toyobo-global.com) and a pair of primers (5′-CATATGCAGTAGTGGCATCA-3′ and 5′-GCTGTAACTTGTCACATCGT-3′) that were designed based on a previous report (*1*).

Next-Generation Sequencing

A cDNA library was prepared from RNA extracted from the feces sample by using the SMARTer Stranded RNA-Seq Kit (Takara-Bio, https://www.takarabio.com). The library

was sequenced by using a NovaSeq 6000 (Illumina, https://www.illumina.com) sequencer. The read sequences were mapped on RaTG13 (GenBank accession no. MN996532), and the Rc-o319 sequence was determined by using CLC genomic workbench version 8.0.1 (QIAGEN, https://www.qiagen.com) software. The sequence was deposited in GenBank (accession no. LC556375).

Phylogenetic Analysis

The nucleotide and amino acid (aa) sequences of sarbecoviruses were aligned by using ClustalW version 2.1(Clustal, https://www.clustal.org). Phylogenetic trees were constructed by performing maximum-likelihood analysis with MEGA version X (*2*), in combination with 500 bootstrap replicates.

Plasmids

We cloned the spike protein (S) genes of Rc-o319 and severe acute respiratory syndrome coronavirus 2 (SARS-CoV-2; 2019-nCoV/Japan/AI/I-004/2020, GenBank accession no. LC521925), with a 19–aa deletion at the C-terminus (pCAGGS-o319-S-del19), into protein expression pCAGGS vectors (pCAGGS-SARS-CoV-2-del19). Earlier reports have suggested that this deletion leads to the efficient production of vesicular stomatitis virus (VSV) pseudotyped with the S gene of SARS-CoV (*3*). We used the SARS-CoV S gene expression plasmid pKS-SARS-St19 (*3*) and the vesicular stomatitis Indiana virus glycoprotein (G)-expression plasmid pCAGGS-VSV-G (*4*). We also constructed angiotensin-converting enzyme 2 (ACE2)-expression plasmids derived from humans (hACE2; GenBank accession no. NM_001371415), greater horseshoe bats (*R. ferrumequinum*) (Rf-ACE2; GenBank accession no. AB297479), and Chinese rufous horseshoe bats (*R. sinicus*) (Rs-ACE2; GenBank accession no. KC881004). The hACE2 cDNA was amplified from RNA isolated from Caco-2 cells by RT-PCR, and the Rf-ACE2 and Rs-ACE2 genes were

artificially synthesized and cloned into pCAGGS plasmids (pCAGGS-hACE2, pCAGGS-Rs-ACE2, or pCAGGS-Rf-ACE2, respectively).

Because fresh RNA samples from *R. cornutus* bats were not available, the ACE2 gene of *R. cornutus* (Rc-ACE2) was amplified from the exon regions of genomic DNA extracted from the kidney of a bat carcass (GenBank accession no. LC564973) and cloned into the pCAGGS vector that was designated as pCAGGS-Rc-ACE2. We also prepared a chimeric bat ACE2 (referred to as Rc/Rf chimera), which consisted of the S interaction domain of Rc-ACE2 (aa positions 15–116) and the remaining region from Rf-ACE2, to form the expression plasmid pCAGGS-Rc/Rf-ACE2.

Western Blot Analysis

HEK293T cells were transfected with pCAGGS-RcACE2, pCAGGS-hACE2, pCAGGS-RfACE2, pCAGGS-RsACE2, pCAGGS-Rc/RfACE2, or empty pCAGGS vectors. One day after transfection, the cells were lysed with SDS sample buffer and subjected to western blotting by using rabbit anti-ACE2 antibody (Abcam, https://www.abcam.com).

Production of VSV-Pseudotyped Virus

VSVΔG*-GFP, which expresses a GFP reporter gene instead of the viral G gene, was used to produce VSV pseudotyped with S genes from sarbecoviruses, as described in earlier reports (*3*,*5*). HEK293T cells seeded on 6-well plates were transfected with 2 μg of either pCAGGS-o319-S-del19, pCAGGS-SARS-CoV-2-del19, pKS-SARS-St19, or control pCAGGS-VSV-G. At 24 h post-transfection, the cells were infected with VSVΔG*-GFP and incubated for 24 h. The culture fluid was collected, centrifuged, filtered through a 0.45-μm filter to remove cells and cell debris, and stored at –80°C until use. The pseudotyped viruses with Rc-o319 S were designated as VSV-Rc-o319, for SARS-CoV S were designated VSV-SARS, for SARS-CoV-2 S were designated VSV-SARS-2, and for VSV-G were designated VSV-VSV-G. The viruses pseudotyped with sarbecovirus S proteins were incubated with the

anti-VSV-G neutralizing antibody I1 (*6*) for 30 min at 21°C to eliminate the remaining VSVΔG*-GFP.

Cell Entry Assay

HEK293T cells seeded on 24-well plates were transfected with 0.5 μg of either pCAGGS-Rc-ACE2, pCAGGS-Rf-ACE2, pCAGGS-Rs-ACE2, pCAGGS-Rc/Rf-ACE2, pCAGGS-hACE2, or control empty pCAGGS plasmids, and incubated for 24 h. Each pseudotyped virus (200 μL) was used to inoculate each ACE2-expression cell culture. After incubation at 37°C for 1 h, the cells were washed once with Opti-MEM (Thermo Fisher Scientific, https://www.thermofisher.com) and incubated with Opti-MEM at 37°C for 20 h. The number of GFP-positive cells within 1 microscopic field (3.1 mm^2) was counted under an Axio Vert.A1 fluorescent microscope (Carl Zeiss, https://www.zeiss.com). The virus titers were determined as the number of GFP-positive cells per well in a 24-well plate (1.9 cm^2), calculated for 5 microscopic fields. The virus titers are expressed in terms of the average values with standard deviations from 3 independent experiments.

Cell Fusion Assay

HEK293T cells were cotransfected with 1 of the S-expression plasmids (pCAGGS-o319-S-del19, pCAGGS-SARS-CoV-2-del19, or pKS-SARS-St19) and 1 of the ACE2-expression plasmids (pCAGGS-Rc-ACE2, pCAGGS-Rf-ACE2, pCAGGS-Rs-ACE2, or pCAGGS-hACE2), with or without the TMPRSS2-expression plasmid, and a fluorescent reporter Venus-expression plasmid (*7*) for the convenient visualization of fused cells under a fluorescence microscope. After transfection, the cells were incubated at 37°C for 24 h. The fused cells were observed under an Axio Vert.A1 fluorescence microscope (Carl Zeiss). As a control, we confirmed that no appreciable syncytium was observed in HEK293T cells transfected with each S-expression plasmid, which indicates that endogeneous hACE2 in these cells did not affect the results of the cell fusion assay.

References

1. Suzuki J, Sato R, Kobayashi T, Aoi T, Harasawa R. Group B betacoronavirus in rhinolophid bats, Japan. J Vet Med Sci. 2014;76:1267–9. PubMed https://doi.org/10.1292/jvms.14-0012

2. Kumar S, Stecher G, Li M, Knyaz C, Tamura K. MEGA X: Molecular evolutionary genetics analysis across computing platforms. Mol Biol Evol. 2018;35:1547–9. PubMed https://doi.org/10.1093/molbev/msy096

3. Fukushi S, Mizutani T, Saijo M, Matsuyama S, Miyajima N, Taguchi F, et al. Vesicular stomatitis virus pseudotyped with severe acute respiratory syndrome coronavirus spike protein. J Gen Virol. 2005;86:2269–74. PubMed https://doi.org/10.1099/vir.0.80955-0

4. Murakami S, Horimoto T, Mai Q, Nidom CA, Chen H, Muramoto Y, et al. Growth determinants for H5N1 influenza vaccine seed viruses in MDCK cells. J Virol. 2008;82:10502–9. PubMed https://doi.org/10.1128/JVI.00970-08

5. Takada A, Robison C, Goto H, Sanchez A, Murti KG, Whitt MA, et al. A system for functional analysis of Ebola virus glycoprotein. Proc Natl Acad Sci U S A. 1997;94:14764–9. PubMed https://doi.org/10.1073/pnas.94.26.14764

6. Iwasa A, Shimojima M, Kawaoka Y. sGP serves as a structural protein in Ebola virus infection. J Infect Dis. 2011;204:S897–903. PubMed https://doi.org/10.1093/infdis/jir313

7. Nagai T, Ibata K, Park ES, Kubota M, Mikoshiba K, Miyawaki A. A variant of yellow fluorescent protein with fast and efficient maturation for cell-biological applications. Nat Biotechnol. 2002;20:87–90. PubMed https://doi.org/10.1038/nbt0102-87

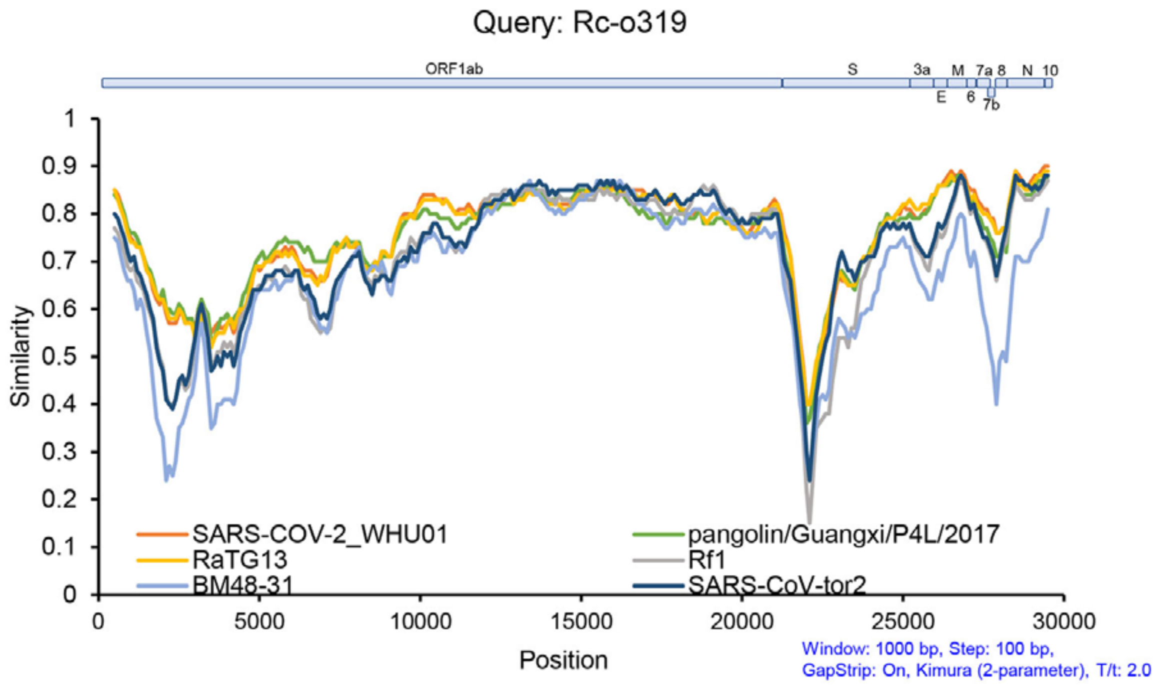

Appendix Figure 1. Similarity plot analysis of sarbecovirus Rc-o319 sequenced from little Japanese horseshoe bats (*Rhinolophus cornutus*) and genetically related to human SARS-CoV-2, Japan. Full-length genome sequence of Rc-o319 was used as query. Representative sequences from sarbecoviruses were used as references. ORF1ab, open reading frame 1ab; SARS-CoV-2, severe acute respiratory syndrome coronavirus 2.

```
R.cornutus        1:MSGSSWLLLSLVAVTAAQSTTEDEAKKFLNDFNSEAENLTYQSSLASWDYNTNISDENVQ 60
R.ferrumequinum   1:MSGSSWLLLSLVAVTAAQSTTEDLAKKFLDDFNSEAENLSHQSSLASWEYNTNISDENVQ 60
R.sinicus         1:MSGSSWLLLSLVAVTTAQSTTEDEAKMFLDKFNTKAEDLSHQSSLASWDYNTNINDENVQ 60
human             1:MSSSSWLLLSLVAVTAAQSTIEEQAKTFLDKFNHEAEDLFYQSSLASWNYNTNITEENVQ 60
                    **.************.****.*. ** **. ** .** *  ******* ***** .****

R.cornutus       61:KMDEAGAKWSAFYEEQSKIAKNYPLEEIQTDIVKRQLQILQQSGSPVLSEDKSKRLNSIL 120
R.ferrumequinum  61:KMDEAGAKWSDFYEKQSKLAKNFSLEEIHNDTVKLQLQILQQSGSPVLSEDKSKRLNSIL 120
R.sinicus        61:KMDEAGAKWSAFYEEQSKLAKNYSLEQIQNVTVKLQLQILQQSGSPVLSEDKSKRLNSIL 120
human            61:NMNNAGDKWSAFLKEQSTLAQMYPLQEIQNLTVKLQLQALQQNGSSVLSEDKSKRLNTIL 120
                    .*..**.***.*...**..*... *..*.. .**.***.***.**.***********.**

R.cornutus      121:NAMSTIYSTGKVCKPNNPQECLLLEPGLDNIMGTSKDYHERLWAWEGWRAEVGKQLRPLY 180
R.ferrumequinum 121:NAMSTIYSTGKVCKPNNPQECLLLEPGLDNIMGTSKDYNERLWAWEGWRAEVGKQLRPLY 180
R.sinicus       121:NAMSTIYSTGKVCKPNKPQECLLLEPGLDNIMGTSKDYNERLWAWEGWRAEVGKQLRPLY 180
human           121:NTMSTIYSTGKVCNPDNPQECLLLEPGLNEIMANSLDYNERLWAWESWRSEVGKQLRPLY 180
                    *.***********.*..***********..**..*.**.*******.**.**********

R.cornutus      181:EEYVVLKNEMARGYHYEDYGDYWRRDYETEESSGPGYSRDQLMKDVDRIFTEIKPLYEHL 240
R.ferrumequinum 181:EEYVVLKNEMARGYHYEDYGDYWRRDYETEGSPDLEYSRDQLIKDVERIFAEIKPLYEQL 240
R.sinicus       181:EEYVVLKNEMARGYHYEDYGDYWRRDYETEESPGPGYSRDQLMKDVERIFTEIKPLYEHL 240
human           181:EEYVVLKNEMARANHYEDYGDYWRGDYEVNGVDGYDYSRGQLIEDVEHTFEEIKPLYEHL 240
                    ************..**********.***.. .  .  ***.** .**...* *******.*

R.cornutus      241:HAYVRAKLMDTYPLHISPTGCLPAHLLGDMWGRFWTNLYPLTVPFGQKPNIDVTDEMVKQ 300
R.ferrumequinum 241:HAYVRTKLMDTYPFHISPTGCLPAHLLGDMWGRFWTNLYPLTVPFGQKPNIDVTDAMLNQ 300
R.sinicus       241:HAYVRAKLMDTYPFHISPTGCLPAHLLGDMWGRFWTNLYPLTVPFGQKPNIDVTDEMLKQ 300
human           241:HAYVRAKLMNAYPSYISPIGCLPAHLLGDMWGRFWTNLYSLTVPFGQKPNIDVTDAMVDQ 300
                    *****.***..** .***.*******************.*************** *  *

R.cornutus      301:GWDANRIFKEAEKFFVSVGLPNMTEGFWNNSMLTEPGDGRKVVCHPTAWDLGKGDFRIKM 360
R.ferrumequinum 301:NWDAKRIFKEAEKFFVSIGLPNMTEGFWNNSMLTDPGDGRKVVCHPTAWDLGKGDFRIKM 360
R.sinicus       301:GWDADRIFKEAEKFFVSVGLPNMTEGFWNNSMLTEPGDGRKVVCHPTAWDLGKGDFRIKM 360
human           301:AWDAQRIFKEAEKFFVSVGLPNMTQGFWENSMLTDPGNVQKAVCHPTAWDLGKGDFRILM 360
                     *** ************.******.***.***** **...*.****************.*

R.cornutus      361:CTKVTMEDFLTAHHEMGHIQYDMAYASQPYLLRNGANEGFHEAVGEVMSLSVATPKHLKT 420
R.ferrumequinum 361:CTKVTMEDFLTAHHEMGHIQYDMAYASQPYLLRNGANEGFHEAVGEVMSLSVATPKHLKT 420
R.sinicus       361:CTKVTMEDFLTAHHEMGHIQYDMAYASQPYLLRNGANEGFHEAVGEVMSLSVATPKHLKT 420
human           361:CTKVTMDDFLTAHHEMGHIQYDMAYAAQPFLLRNGANEGFHEAVGEIMSLSAATPKHLKS 420
                    ******.*******************.**.****************.****.*******.

R.cornutus      421:MGLLSPDFREDDETEINFLLKQALNIVGTLPFTYMLEKWRWMVFKGEIPKEEWMKKWWEM 480
R.ferrumequinum 421:MGLLSSDFLEDNETEINFLFKQALNIVGTLPFTYMLEKWRWMVFKGEIPKEEWMKKWWEM 480
R.sinicus       421:MGLLSPDFREDNETEINFLLKQALNIVGTLPFTYMLEKWRWMVFKGEIPKEEWMKKWWEM 480
human           421:IGLLSPDFQEDNETEINFLLKQALTIVGTLPFTYMLEKWRWMVFKGEIPKDQWMKKWWEM 480
                    .****.** **.*******.****.**************************..********

R.cornutus      481:RREIVGVVEPVPHDETYCDPASLFHVANDYSFIRYYTRTIFEFQFHEALCRIAQHNGPLH 540
R.ferrumequinum 481:KRKIVGVVEPVPHDETYCDPASLFHVANDYSFIRYYTRTIFEFQFHEALCRIAKHDGPLH 540
R.sinicus       481:KRKIVGVVEPVPHDETYCDPASLFHVANDYSFIRYYTRTIFEFQFHEALCRIAQHDGPLH 540
human           481:KREIVGVVEPVPHDETYCDPASLFHVSNDYSFIRYYTRTLYQFQFQEALCQAAKHEGPLH 540
                    .* **********************.************...***.****..* * ****

R.cornutus      541:KCDISNSTDAGKKLHQMLSVGKSQAWTKTLEDIVGSRNMDVGPLLRYFEPLYTWLQEQNR 600
R.ferrumequinum 541:KCDISNSTDAGEKLHQMLSVGKSQPWTSVLKDFVGSKNMDVGPLLRYFEPLYTWLTEQNR 600
R.sinicus       541:KCDISNSTDAGKKLHQMLSVGKSQAWTKTLEDIVDSRNMDVGPLLKYFEPLYTWLQEQNR 600
human           541:KCDISNSTEAGQKLFNMLRLGKSEPWTLALENVVGAKNMNVRPLLNYFEPLFTWLKDQNK 600
                    ********.** **..**..***. **  *.. *.. **.*.*** *****.*** .**.

R.cornutus      601:KSYVGWNTDWSPYSDQSIKVRISLKSALGEKAYEWNDNEMYLFRSSVAYAMREYFLKTKN 660
R.ferrumequinum 601:KSFVGWNTDWSPYADQSIKVRISLKSALGEKAYEWNNNEMYLFRSSVAYAMREYFLKTKN 660
R.sinicus       601:KSYVGWNTDWSPYSDQSIKVRISLKSALGENAYEWNDNEMYLFRSSVAYAMREYFLKEKH 660
human           601:NSFVGWSTDWSPYADQSIKVRISLKSALGDKAYEWNDNEMYLFRSSVAYAMRQYFLKVKN 660
                    .* ***.****** ***************..*****.***************.**** *.

R.cornutus      661:QTILFGDENVWVSNLKPRISFNFHVTSPENVSDIIPRSEVEGAIRMSRSRINDAFRLDDN 720
R.ferrumequinum 661:QTILFGEEDVWVSNLKPRISFNFYVTSPRNLSDIIPKPEVEGAIRMSRSRINDAFRLDDN 720
R.sinicus       661:QTILFGAENVWVSNLKPRISFNFHVTSPGNLSDIIPRPEVEGAIRMSRSRINDAFRLDDN 720
human           661:QMILFGEEDVRVANLKPRISFNFFVTAPKNVSDIIPRTEVEKAIRMSRSRINDAFRLNDN 720
                    *.**** * *.*.********** **.* * *****. ***.***************.**

R.cornutus      721:SLEFLGIQPTLGPPYQPPVTIWLIVFGVVMAVVVVGIVVLIITGIRDRRKTDQARSEENP 780
R.ferrumequinum 721:SLEFLGIQPTLGPPYQPPVTIWLIVFGVVMAVVVVGIVVLIITGIRDRRKKDQARSEENP 780
R.sinicus       721:SLEFLGIQPTLGPPYQPPVTIWLIVFGVVMAVVVVGIVVLIITGIRDRRKTDQARSEENP 780
human           721:SLEFLGIQPTLGPPNQPPVSIWLIVFGVVMGVIVVGIVILIFTGIRDRKKKNKARSGENP 780
                    **************.****.**********.*.*****.**.******.* ..***.***

R.cornutus      781:YPSVDLSKGENNPGFQNGDDVQTSF                                   805
R.ferrumequinum 781:YSSVDLSKGENNPGFQNGDDVQTSF                                   805
R.sinicus       781:YSSVDLSKGENNPGFQNGDDVQTSF                                   805
human           781:YASIDISKGENNPGFQNTDDVQTSF                                   805
                    * *.*.***********.*******
```

Appendix Figure 2. Alignment of ACE2 amino acid sequence from *Rhinolophus cornutus*, *R. ferrumequinum*, *R. sinicus,* and humans. Deduced amino acid sequence of *R. cornutus* ACE2 RNA was aligned with those of *R. ferrumequinum, R. sinicus,* and human ACE2. Highlighted yellow region was considered the spike protein binding region of *R. cornutus* ACE2. ACE2, angiotensin-converting enzyme 2.

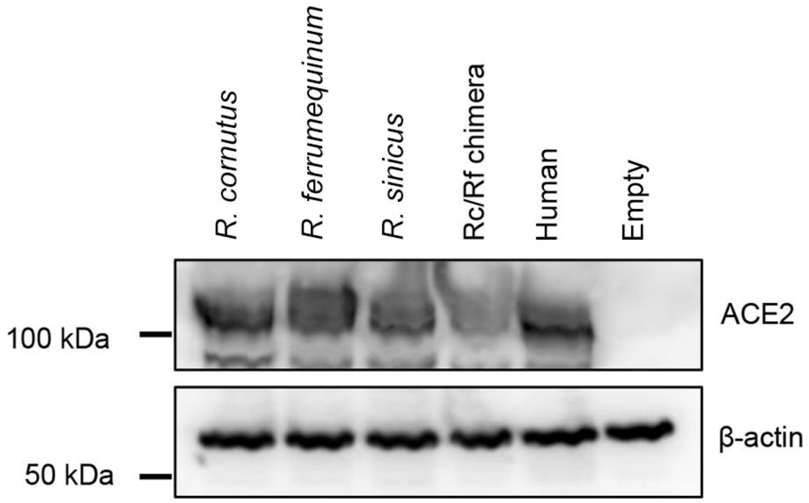

Appendix Figure 3. Western blot analysis showing expression of angiotensin-converting enzyme 2 in transfected cells from bats and humans. HEK293T cells that transiently expressed *Rhinolophus cornutus*, *R. ferrumequinum*, *R. sinicus*, Rc/Rf chimera (spike protein [S] interaction domain from *R. cornutus* and the remaining from *R. ferrumequinum*), or human ACE2. ACE2, angiotensin-converting enzyme 2; HEK293T cells, human embryonic kidney 293T cells.

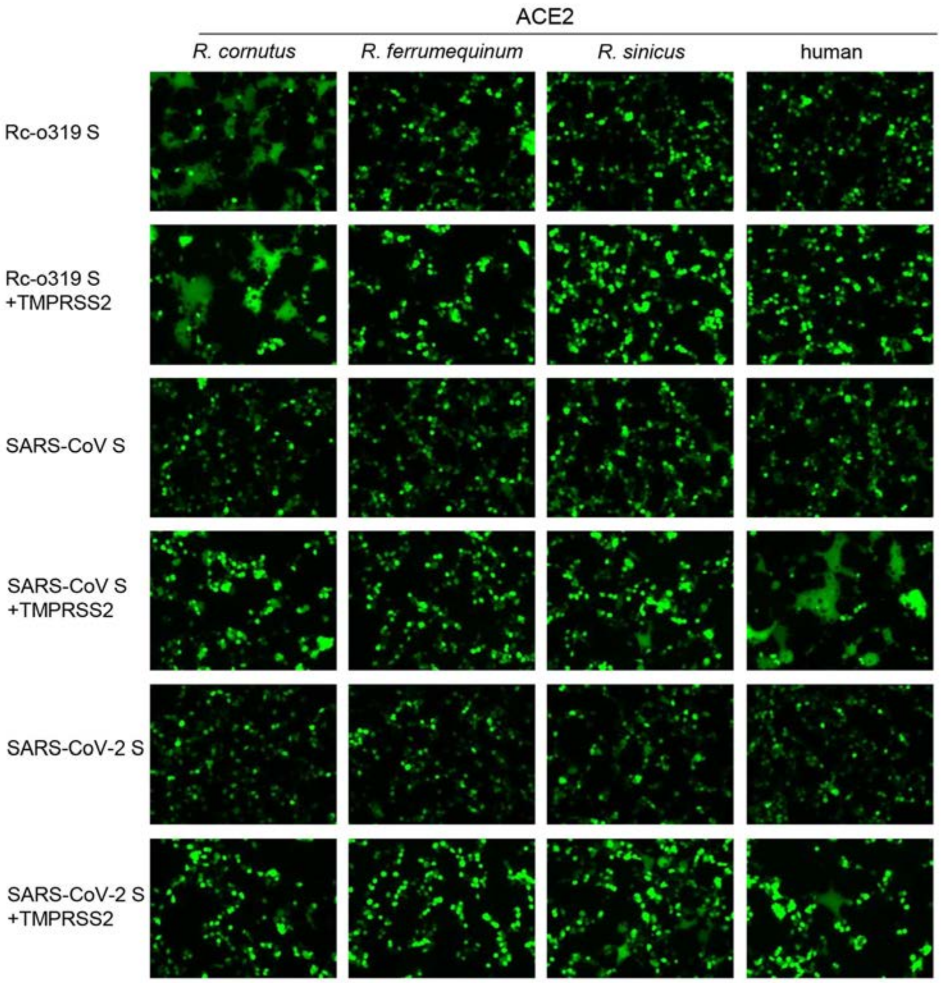

Appendix Figure 4. Results of fusion assay in which HEK293T cells were cotransfected with S-expression plasmids and ACE2 from bat and human sarbecoviruses. S-expression plasmids of Rc-o319, SARS-CoV, or SARS-CoV-2 and expression plasmids of Rc-ACE2, Rf-ACE2, Rs-ACE2, or hACE2, and fluorescent reporter Venus-expression plasmid with and without TMPRSS2-expression plasmid were incubated for 24 h to assess syncytium formation. At 24 h post-transfection, the cells were observed under a fluorescence microscope. ACE2, angiotensin-converting enzyme 2; hACE2, human ACE2; HEK293T cells, human embryonic kidney 293T cells; Rc-o319, sarbecovirus identified in this study; Rc-ACE2, *Rhinolophus cornutus* ACE2; Rf-ACE2, *R. ferrumequinum* ACE2; Rs-ACE2, *R. sinicus* ACE2; S, spike glycoprotein; SARS-CoV, severe acute respiratory syndrome coronavirus; SARS-CoV-2, severe acute respiratory syndrome coronavirus 2; TMPRSS2, transmembrane serine protease 2.
